# Tailless and hypoxia inducible factor-2α cooperate to sustain proangiogenic states of retinal astrocytes in neonatal mice

**DOI:** 10.1242/bio.059684

**Published:** 2023-01-10

**Authors:** Li-Juan Duan, Yida Jiang, Yanhong Shi, Guo-Hua Fong

**Affiliations:** ^1^ Center for Vascular Biology, Department of Cell Biology, University of Connecticut Health Center, Farmington, Connecticut 06030, USA; ^2^ Department of Cell Biology, University of Connecticut Health Center, Farmington, Connecticut 06030, USA; ^3^ Department of Stem Cell Biology and Regenerative Medicine, City of Hope, 1500 East Duarte Road, Duarte, CA 91010, USA

**Keywords:** Oxygen sensing, Hypoxia inducible factor, Retinal angiogenesis, Tailless, Retinal astrocytes

## Abstract

Tailless (TLX, an orphan nuclear receptor) and hypoxia inducible factor-2α (HIF2α) are both essential for retinal astrocyte and vascular development. *Tlx*^−/−^ mutation and astrocyte specific *Hif2α* disruption in *Hif2α^f/f^/GFAPCre* mice are known to cause defective astrocyte development and block vascular development in neonatal retinas. Here we report that TLX and HIF2α support retinal angiogenesis by cooperatively maintaining retinal astrocytes in their proangiogenic states. While *Tlx*^+/−^ and *Hif2α^f/+^/GFAPCre* mice are phenotypically normal, *Tlx*^+/−^/*Hif2α^f/+^/GFAPCre* mice display precocious retinal astrocyte differentiation towards non-angiogenic states, along with significantly reduced retinal angiogenesis. In wild-type mice, TLX and HIF2α coexist in the same protein complex, suggesting a cooperative function under physiological conditions. Furthermore, astrocyte specific disruption of *Phd2* (prolyl hydroxylase domain protein 2), a manipulation previously shown to cause HIF2α accumulation, did not rescue retinal angiogenesis in *Tlx^−/−^* background, which suggests functional dependence of HIF2α on TLX. Finally, the expression of fibronectin and VEGF-A is significantly reduced in retinal astrocytes of neonatal *Tlx^+/−^/Hif2α^f/+^/GFAPCre* mice. Overall, these data indicate that TLX and HIF2α cooperatively support retinal angiogenesis by maintaining angiogenic potential of retinal astrocytes.

## Teaser

**Summary:** Retinal astrocytes lose their ability to support angiogenesis as they mature. Protein–protein interaction between Tlx and HIF-2α maintains retinal astrocytes in their proangiogenic progenitor and immature states.

## INTRODUCTION

In mice, retinal vascular development begins at birth, with nascent vascular sprouts emerging from central vessels at the edge of the optic nerve head (ONH). During the subsequent week or so, the vascular network expands radially, reaching the retinal periphery by postnatal day (P) 7-8 (Duan et al., 2017; Fruttiger, 2002; Gariano et al., 1996; Gerhardt et al., 2003; Hellström et al., 2007; Stone and Dreher, 1987). Besides endothelial cells (ECs), astrocytes are also critical for retinal vascular development (Duan et al., 2017; Gariano et al., 1996; Paisley and Kay, 2021; Perelli et al., 2021; Stone and Dreher, 1987; Tao and Zhang, 2014).

Retinas in newborn mice lack differentiated astrocytes outside the ONH. Instead, the central half of the superficial retina is populated with astrocyte progenitors, whereas the more peripheral half is still vacant. As retinal vascularization progresses, astrocyte progenitors differentiate towards more mature states (Duan et al., 2017), beginning near the ONH and advancing towards the retinal periphery. Ahead of the vascular front, most astrocytes in the avascular area exist as progenitors in the first day of neonatal life, and as immature (partially differentiated) astrocytes after P1 (Duan et al., 2017; Ling et al., 1989). Both progenitors and immature astrocytes migrate rapidly, reaching retinal periphery by P3-P4. Furthermore, these early astrocytes are proliferative and proangiogenic, whereas fully differentiated (mature) astrocytes are static and non-angiogenic (Duan et al., 2017; Ling et al., 1989).

Retinal astrocytes can be identified based on their expression of several proteins, such as paired box 2 (PAX2, a transcription factor), glial fibrillary acidic protein (GFAP, a cytoskeletal protein in retinal astrocytes), and PDGF receptor-α (PDGFRα) (Dorrell et al., 2002; Fruttiger, 2002; Mi and Barres, 1999; Stanke et al., 2010). While these proteins are expressed in multiple cell types in different organs, they are specific to the astrocytic cell lineage within early neonatal retinas (i.e. during angiogenesis of superficial vasculature).

PAX2 and GFAP display distinct temporal regulation during astrocyte development. PAX2 is expressed at all stages from progenitors to mature astrocytes, but the level of expression gradually decreases as astrocytes become more mature (Duan et al., 2017). On the other hand, GFAP is absent in progenitors but is strongly upregulated in mature astrocytes. Because all retinal astrocytes express PAX2 (although at reduced levels in mature astrocytes), the GFAP/PAX2 ratio in a selected retinal area is a reliable barometer of local astrocyte differentiation, with higher ratio indicating more advanced differentiation towards maturity (Duan et al., 2017).

At the molecular level, retinal astrocyte differentiation is controlled, at least in part, by oxygen sensing mechanisms (Bruick and McKnight, 2001; Carmeliet et al., 1998; Duan and Fong, 2019; Duan et al., 2017, 2014; Epstein et al., 2001; Ivan et al., 2002; Maxwell et al., 1999; Perelli et al., 2021; Wang and Semenza, 1995; West et al., 2005). Oxygen sensing and hypoxia responses are ubiquitous processes in different types of cells, and are mediated by prolyl hydroxylase domain proteins (PHDs, also known as Egg Laying Nine or EGLN) and hypoxia inducible factor (HIF)-α proteins (Bruick and McKnight, 2001; Carmeliet et al., 1998; Epstein et al., 2001; Ivan et al., 2002; Jiang et al., 2021; Maltepe et al., 1997; Peng et al., 2000; Tian et al., 1998, 1997; Wang et al., 1995). In well oxygenated tissues, PHDs catalyze prolyl hydroxylation of HIF-α proteins, labeling them for pVHL and E3 ligase dependent polyubiquitination and proteasomal degradation. Under hypoxic conditions, PHD activities are suppressed due to the shortage of O_2_ as a substrate, allowing HIF-α proteins to accumulate to high levels (Epstein et al., 2001; Min et al., 2002).

In retinal astrocytes of neonatal mice, PHD2 and HIF2α have been identified as the main PHD and HIF family members mediating oxygen sensing and hypoxia signaling (Duan and Fong, 2019; Duan et al., 2014). In *Phd2^f/f^/GFAPCre* mice, astrocyte specific *Phd2* disruption led to the accumulation of HIF2α protein and suppression of astrocyte maturation, whereas astrocyte specific *Hif2α* deletion accelerated retinal astrocyte maturation and blocked retinal angiogenesis (Duan and Fong, 2019; Duan et al., 2017, 2014; Perelli et al., 2021).

Tailless (TLX, also known as NR2E1 for nuclear receptor subfamily 2 group E member 1) (Shi et al., 2004; Yu et al., 1994) is essential for retinal vascular development (Miyawaki et al., 2004; Uemura et al., 2006). Within the superficial retinal tissue, which is where astrocytic and retinal vascular development takes place in the first week after birth, TLX is specifically expressed in retinal astrocytes but not in endothelial cells (Miyawaki et al., 2004; Uemura et al., 2006). Furthermore, within the astrocyte population, TLX expression is limited to the astrocytes in the avascular area, whereas astrocytes in vascularized areas are TLX negative (Uemura et al., 2006).

In *Tlx^−/−^* mice, astrocyte migration is suppressed, and vascular development is completely blocked, most likely because of defective fibronectin deposition from TLX deficient retinal astrocytes (Miyawaki et al., 2004; Uemura et al., 2006). The avascular retinal phenotypes in *Tlx^−/−^* mice are very similar to those in *Hif2α^f/f^/GFAPCre* mice (Duan et al., 2014; Uemura et al., 2006). These similarities suggested that TLX and HIF2α might play related roles in retinal astrocytes and vascular development and prompted us to investigate the potential interactions between them. Data presented in this communication support a mechanism wherein the two factors collaboratively maintain proangiogenic states of retinal astrocytes and support retinal angiogenesis in a fibronectin and VEGF-A dependent pathway.

## RESULTS AND DISCUSSION

### Precocious retinal astrocyte differentiation in *Tlx^+/−^/Hif2α^f/+^/GFAPCre* mice

We evaluated retinal astrocyte differentiation at P0.5 (i.e. early afternoon in the day of birth). At P0.5, retinas are essentially avascular, with the exception of a few nascent sprouts in the immediate vicinity of the ONH. This condition is advantageous for analyzing astrocyte differentiation because in avascular retinal tissues, it can be assumed that the observed astrocyte phenotypes most likely reflect inherent astrocyte properties, rather than secondary effects due to interaction with blood vessels.

*Tlx^+/−^/Hif2α^f/+^/GFAPCre* mice were generated by crossing *Tlx^+/−^* and *Hif2α^f/f^/GFAPCre* mice. The particular line of *GFAPCre* transgenic mice used in this study (Jax stock number 004600) (Zhuo et al., 2001) has been demonstrated to drive robust Cre expression in retinal astrocyte progenitors (Duan et al., 2014; Perelli et al., 2021), despite the fact that the endogenous *Gfap* promoter is active only in more mature astrocytes.

**Fig. 1.**
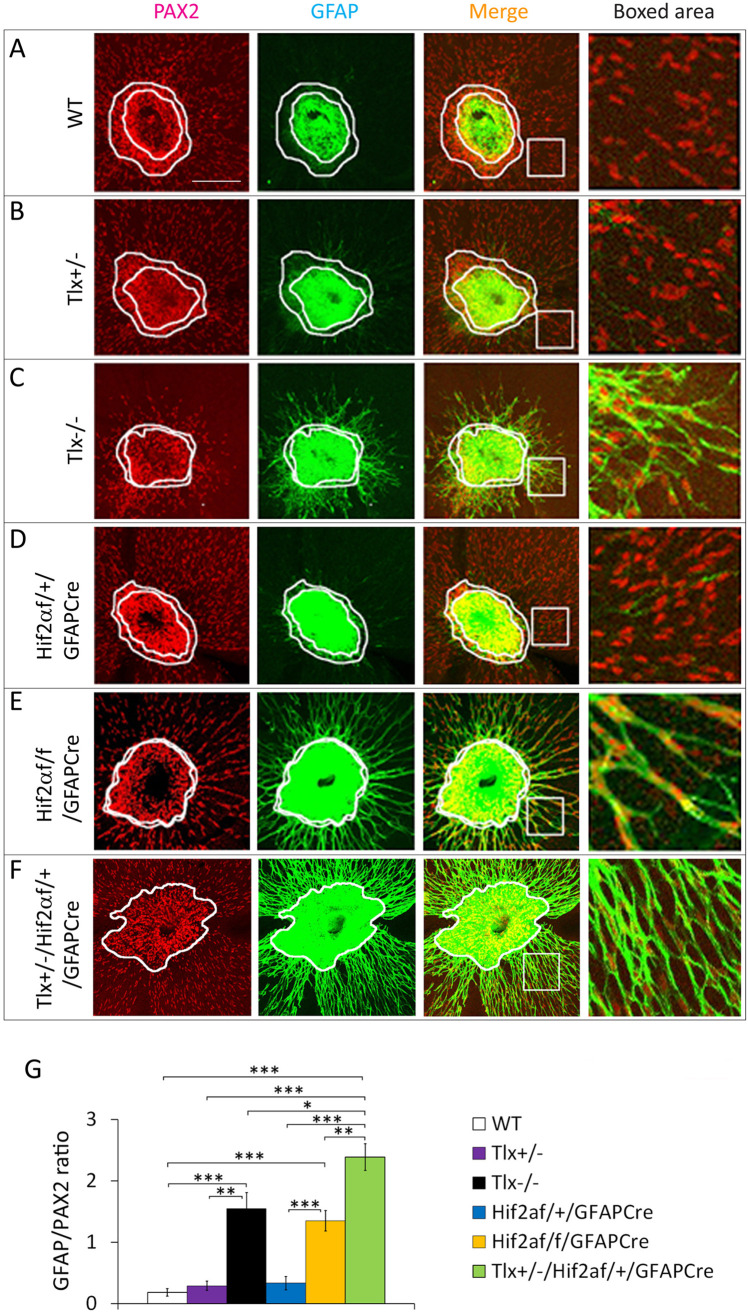
**Accelerated retinal astrocyte maturation in *Tlx^+/−^*/*Hif2α^f/+^/GFAPCre* mice.** (A-F) Retinal confocal images from P0.5 mice, double-stained with anti-PAX2 and anti-GFAP. Inner and outer white contours mark the GFAP^+^ core and GFAP^−^/PAX2^+^ rim of the ONH, respectively. Note that the GFAP^−^/PAX2^+^ rim is extremely narrow (or virtually non-existent) in *Tlx^−/−^*, *Hif2α^f/f^/GFAPCre*, and *Tlx^+/−^*/*Hif2α^f/f^/GFAPCre* mice. Scale bar: 200 µm. (G) Quantification of GFAP/PAX2 ratio. Areas chosen for quantification are indicated by white boxes. *n*=6 to 8. **P<0.01; ***P<0.001.

Astrocyte differentiation was analyzed by whole-mount immunofluorescence (IF) staining with anti-GFAP and anti-PAX2. IF-stained retinas were flat-mounted and examined by confocal imaging (Fig. 1). In several groups of control mice, including WT, *Tlx^+/−^*, and *Hif2α^f/+^/GFAPCre* mice, GFAP^+^ cells were restricted to the core area of the ONH (Fig. 1A,B,D). GFAP^+^ cells at this location are part of the optic nerve, but not a source for retinal astrocyte development (Duan et al., 2017; Ling et al., 1989; Mi and Barres, 1999). By contrast, GFAP^−^/PAX2^+^ cells at the rim of the ONH are retinal astrocyte progenitors (Fig. 1A, B, and D, between the two white lines), capable of migrating onto and populating superficial retinal tissues (Duan et al., 2017; Ling et al., 1989; Mi and Barres, 1999). In WT, *Tlx^+/−^*, and *Hif2α^f/+^/GFAPCre* mice, the GFAP^−^/PAX2^+^ cells form a high density ring in the ONH rim, and are also abundantly present in the central half of superficial retinal tissues (Fig. 1A,B,D). On the other hand, GFAP^+^ cells were scarce beyond the GFAP^+^ ONH core (Fig. 1A,B,D).

In P0.5 *Tlx^−/−^* mice, the uniformly GFAP^+^ staining in the ONH expanded into the rim region, leaving only a negligibly narrow ring of GFAP^−^/PAX2^+^ cells (Fig. 1C). Furthermore, GFAP^+^ cells were readily detectable outside the ONH, suggesting that retinal astrocytes are already differentiating towards maturity at or soon after birth. Precocious maturation of retinal astrocytes was previously unknown in *Tlx^−/−^* mice. Notably, this phenotype is similar to that in *Hif2α^f/f^/GFAPCre* mice, which was reported by us in a previous publication (Duan et al., 2014), but an image is also included in Fig. 1E for comparison to *Tlx^−/−^* mice.

Although *Tlx^+/−^ and Hif2α^f/+^/GFAPCre* mice were phenotypically normal, when the two targeted alleles were combined in *Tlx^+/−^/Hif2α^f/+^/GFAPCre* mice, retinal astrocytes differentiated towards maturity as early as P0.5. The GFAP^+^ region in the ONH expanded at the expense of the PAX2^+^ rim, and GFAP expression was strongly upregulated in retinal astrocytes (Fig. 1F).

To quantify retinal astrocyte differentiation, we calculated the ratio between GFAP^+^ and PAX2^+^ pixel values (Fig. 1G). The GFAP/PAX2 ratio in the peri-ONH area differed vastly among different mouse lines. In *Tlx^+/−^* and *Hif2α^f/+^/GFAPCre* mice, this ratio was similar to wild-type (WT) mice (all being close to 0), confirming that the majority of retinal astrocytes were in undifferentiated progenitor states in these mice. In *Tlx^−/−^*, *Hif2α^f/f^/GFAPCre*, and *Tlx^+/−^/Hif2α^f/+^/GFAPCre* mice, the GFAP/PAX2 ratio was significantly higher than in WT mice.

### Defective retinal vascular development in mice partially deficient for both TLX and HIF2α

**Fig. 2.**
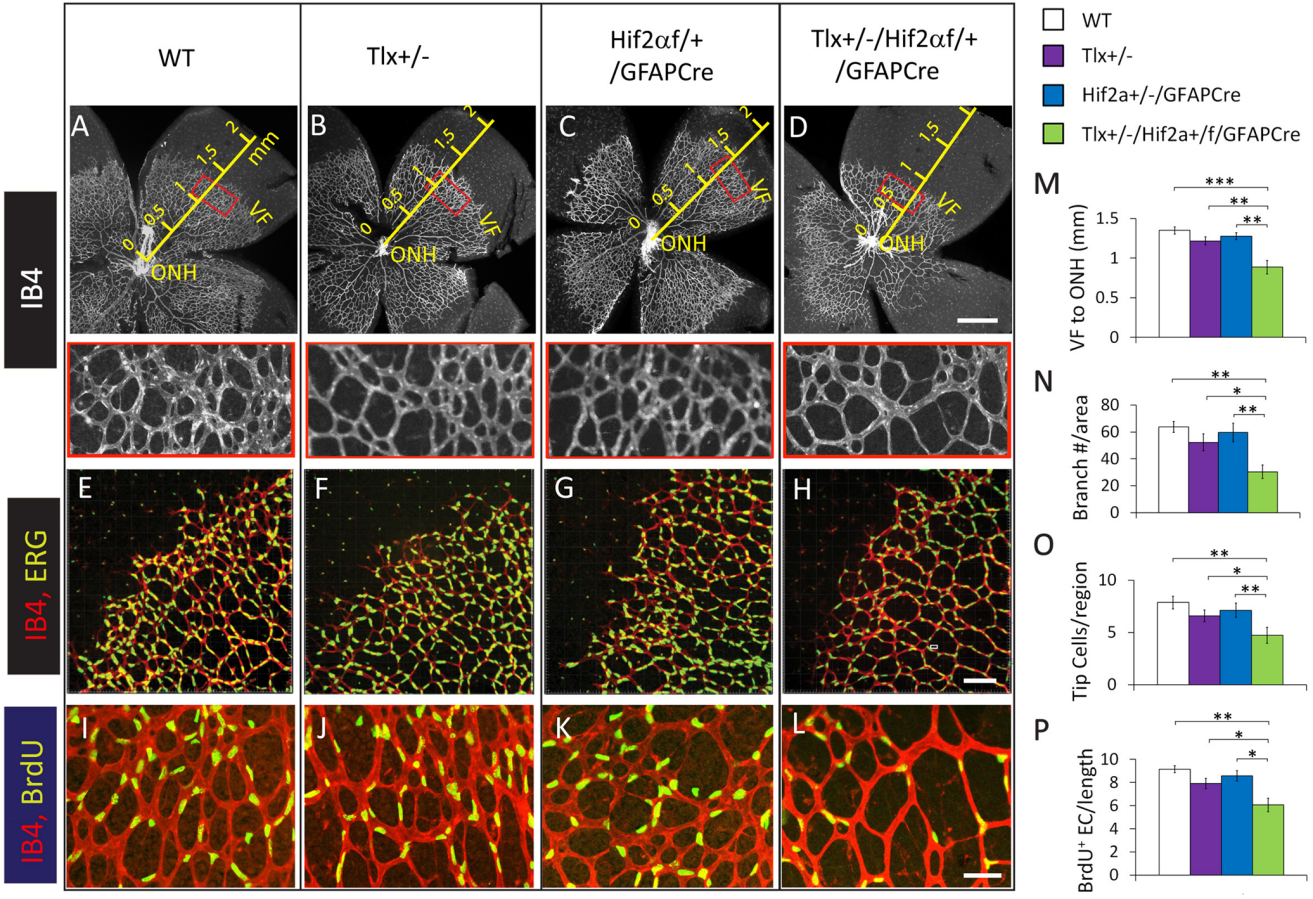
**Compromised vascular morphogenesis in *Tlx^+/−^*/*Hif2α^f/+^/GFAPCre* mice.** (A-D) Confocal images of IB_4_-stained P6 retinas. Red rectangles (expanded below the main images) indicate approximate locations where quantification of vascular branching points was carried out. Scale bar: 500 µm; VF, vascular front; ONH, optic nerve head. (E-H) Z-stack images (8 µm) of P6 retinas double-stained with IB_4_ and anti-ERG. IB_4_^+^ vascular tip cells are located at the VF, bear filopodia extensions, and contain large ERG^+^ nuclei. Scale bar: 100 µm. (I-L) EC proliferation assay by BrdU labeling. Images were from areas near the VF. (M) Distance (mm) between VF and ONH. (N) Number of microvascular branches per 0.08 mm^2^ area at locations marked by red rectangles (A-D). (O) Number of tip cells per 0.5 mm of vascular front. (P) Number of BrdU^+^ nuclei per 0.5 mm length of vascular structure. *n*=6 to 8. *P<0.05; **P<0.01; ***P<0.001.

Retinal vascular development was examined at P6 by whole-mount staining with isolectin B_4_ (IB_4_), followed by flat-mounting and confocal imaging (Fig. 2). In WT, *Tlx^+/−^*, and *Hif2α^f/+^/GFAPCre* mice, the vascular front (VF) typically traveled beyond midway between the ONH and the retinal periphery. In *Tlx^+/−^/Hif2α^f/+^/GFAPCre* mice, the VF advanced significantly less (Fig. 2A-D,M).

Vascular branching morphogenesis was quantified as the number of microvascular branching points per area (also referred to as microvascular density). Areas just behind the vascular front (red rectangular areas in Fig. 2A-D) were chosen for this analysis, because microvascular density at this location most directly reflects branching morphogenesis, rather than vascular pruning. As summarized in Fig. 2N, microvascular density in *Tlx^+/−^/Hif2α^f/+^/GFAPCre* mice was significantly reduced relative to WT, *Tlx^+/−^*, and *Hif2α^f/+^/GFAPCre* mice.

Vascular tip cells were identified according to a published method (Yanagida et al., 2020), which identifies EC nuclei by anti-ERG (*Ets*-related gene)-IF staining (Fig. 2E-H). Briefly, P6 retinas were double-stained with IB_4_ and anti-ERG (*Ets*-related gene), and Z-stack confocal images were constructed at 8 µm. Tip cells were identified as IB_4_^+^ cells containing large ERG^+^ nuclei, being located at the vascular front, and extending filopodia towards avascular areas (Fig. 2E-H). In our hands, microglial cells were also ERG^+^. However, microglial cells in neonatal retinas have very small nuclei, and most of them are located in the avascular area, away from the vascular front (Fig. 2E-H). Quantification of tip cells yielded significantly reduced numbers in *Tlx^+/−^/Hif2α^f/+^/GFAPCre* mice relative to WT, *Tlx^+/−^*, and *Hif2α^f/+^/GFAPCre* mice (Fig. 2O).

Analysis of EC proliferation was carried out at P6. Briefly, mice were injected with 5′ bromo-2′ deoxyuridine (BrdU) intraperitoneally, an hour before euthanasia, and whole-mount retinas were double-stained with IB_4_ and anti-BrdU antibody (Fig. 1I-L). Proliferating ECs were quantified in retinal areas just behind the vascular front. As summarized in Fig. 2P, *Tlx^+/−^/Hif2α^f/+^/GFAPCre* mice had significantly less proliferating ECs, calculated as the number of BrdU^+^ nuclei per 500 µm length of IB_4_^+^ microvessels.

To evaluate EC apoptosis, a positive control was established by treating mice with 75% oxygen for 14 h between P7 and P8. Whole-mount retinas from oxygen-treated mice were subject to IB_4_ and anti-Caspase (cleaved) IF staining, which revealed numerous ECs positive for cleaved Caspase 3. However, apoptotic retinal ECs were detected only sporadically in all mice housed under room air at all times (Fig. S1). These findings indicate that TLX and HIF2α double insufficiency has little impact on the survival of retinal ECs.

### Cooperative functions between TLX and HIF2α

**Fig. 3.**
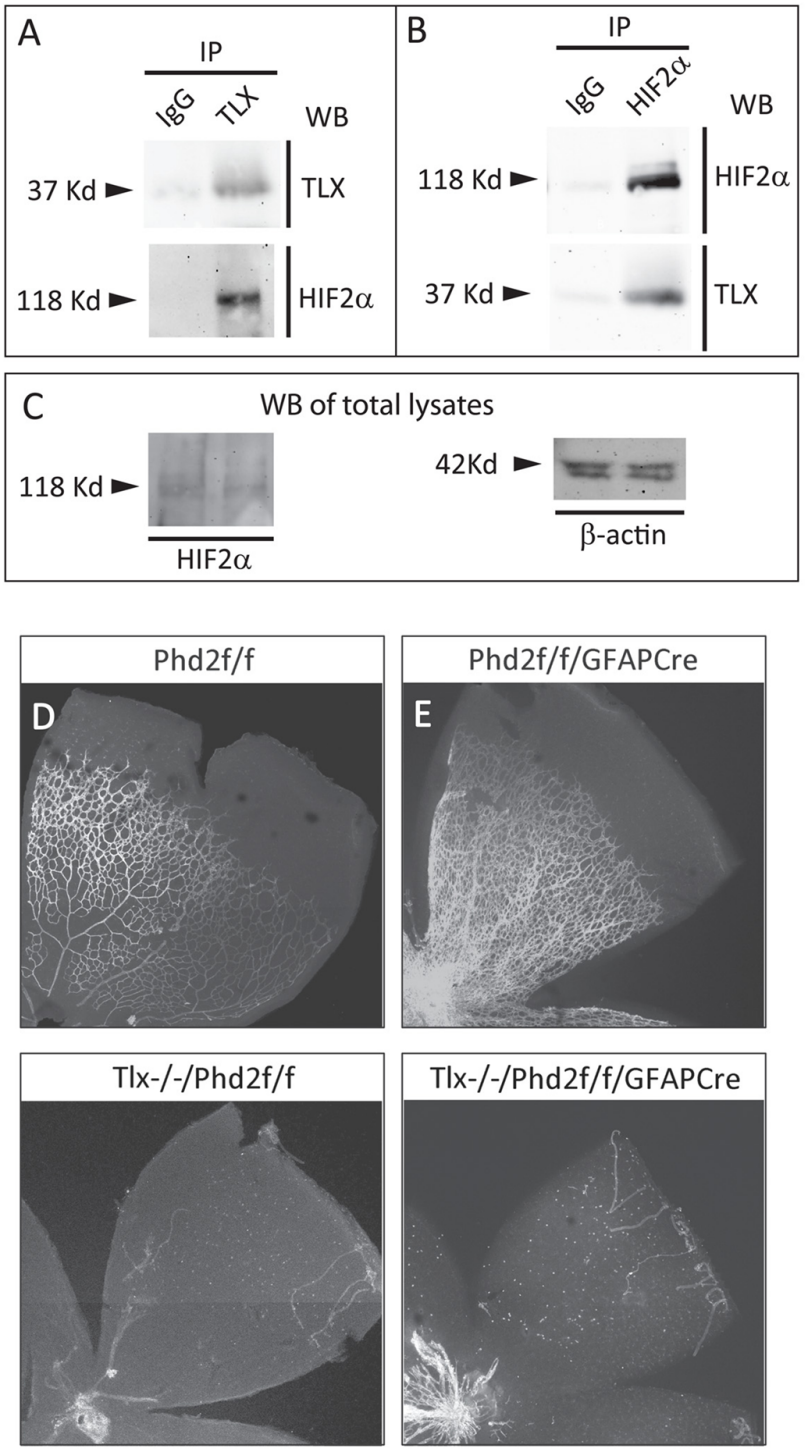
**Cooperative role of TLX and HIF2α.** (A-C) Immunoprecipitation (IP) and Western blotting (WB) of retinal lysates from P4 WT mice. (A) Anti-TLX IP followed by WB using indicated antibodies. HIF2α was co-immunoprecipitated by anti-TLX but not by nonspecific IgG; (B) Anti-HIF2α IP, followed by WB analysis. TLX was specifically co-immunoprecipitated by anti-HIF2α. (C) WB of total retinal lysates. Each experiment was repeated in three independent pools of retinal lysates (retinas from three mice per pool; nine mice for three independent pools). (D-G) Confocal images of IB_4_-stained retinas from P7 mice. The analysis of retinal vascular phenotypes in *Phd2^f/f^ and Phd2^f/f^/GFAPCre* mice have been published (Duan and Fong, 2019), but images in D and E are included as controls for F and G, respectively, and to indicate that the lack of retinal vascular development in *Tlx^−/−^*/*Phd2^f/f^* and *Tlx^−/−^*/*Phd2^f/f^/GFAPCre* mice is because of TLX deficiency but not because of any genetic modification in the *Phd2* gene. Retinal vascular phenotypes in F and G were reproducible in all of four to five mice observed per genotype. Scale bar: 500 µm.

Abnormal astrocyte and vascular phenotypes in neonatal *Tlx^+/−^/Hif2α^f/+^/GFAPCre* mice raised the possibility that in normal neonatal mice, TLX and HIF2α play cooperative roles through protein–protein interactions. Therefore, we investigated whether TLX and HIF2α exist in the same protein complex in WT mouse retinas by co-immunoprecipitation (Fig. 3A-C). Briefly, retinal lysates were prepared from WT mice at P4, and incubated with anti-TLX or anti-HIF2α antibodies, respectively. Immunoprecipitation with anti-TLX pulled down HIF2α that was easily detectable in anti-HIF2α Western blots (Fig. 3A). In a complementary experiment, immunoprecipitation was carried out with anti-HIF2α and TLX was detected by anti-TLX Western blotting (Fig. 3B). These data demonstrate that TLX and HIF2α co-exist in the same protein complex in normal retinal tissues.

While the coexistence of TLX and HIF2α in the same protein complex suggests that they might exert cooperative functions through protein–protein interaction, an alternative possibility might be that TLX facilitates HIF2α stabilization (Zeng et al., 2012). If HIF2α stabilization is all that is needed, it might be possible to bypass the need for TLX by stabilizing HIF2α through a TLX-independent mechanism. To test this possibility, we generated *Tlx^−/−^*/*Phd2^f/f^/GFAPCre* mice by stepwise crossing between *Tlx^+/−^* and *Phd2^f/f^/GFAPCre* mice. High efficiency *Phd2* deletion and robust HIF2α stabilization and accumulation have been previously demonstrated in *Phd2^f/f^/GFAPCre* mice (Duan and Fong, 2019). By P7, retinas in *Tlx^−/−^*/*Phd2^f/f^/GFAPCre* mice remained essentially avascular, similar to those in *Tlx^−/−^* mice (Fig. 3D-G). This outcome suggests that HIF2α stabilization in the absence of TLX is insufficient to support retinal vascular development.

### Diminished fibronectin and VEGF-A expression in retinal astrocytes of *Tlx^+/−^/Hif2α^f/+^/GFAPCre* mice

It is known that both retinal ECs and astrocytes express fibronectin (FN) in neonatal mice (Uemura et al., 2006). Furthermore, astrocyte-derived FN is important for retinal vascular development (Stenzel et al., 2011). However, FN expression in the progenitors of retinal astrocytes has not been directly documented. To examine FN expression in retinal astrocyte progenitors in WT mice, we performed double IF-staining with anti-FN and anti-PDGFRα at P0.5 (Fig. S2). The FN^+^ and PDGFRα^+^ signals form similar patterns; both being limited to the central half of the retina (Fig. S2A). Furthermore, FN^+^ signals were closely associated with PDGFRα^+^ astrocytes (Fig. S2B). Because essentially all retinal astrocytes are at the progenitor stage in WT P0.5 mice, these data indicate that astrocyte progenitors produce abundant amounts of fibronectin.

**Fig. 4.**
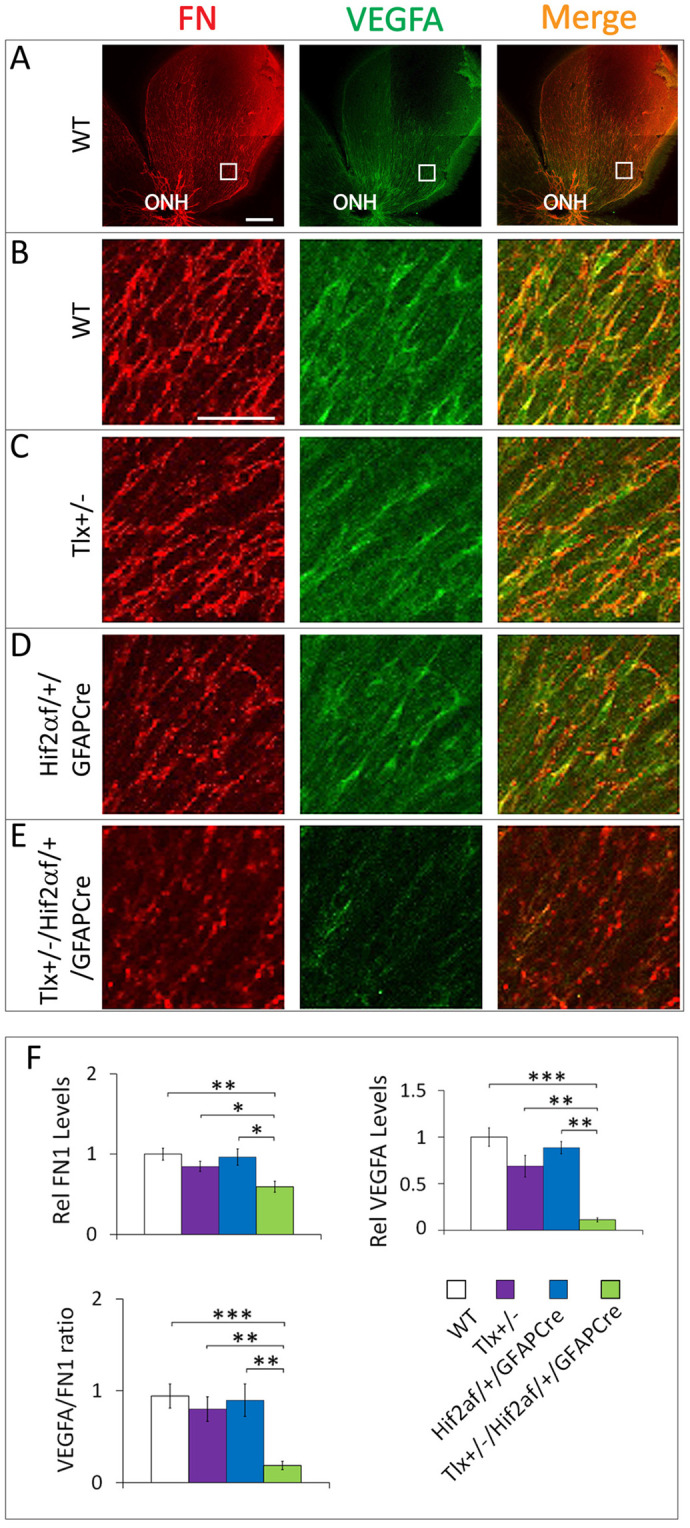
**Diminished FN and VEGF-A expression in superficial retinal tissues of *Tlx^+/−^*/*Hif2α^f/f^/GFAPCre*.** (A) Low magnification (tiled) confocal retinal images of a P0.5 WT mouse, taken after anti-FN and anti-VEGF-A IF-staining and flat-mounting. These images are included to indicate the approximate location (white boxes) where all high magnification images were taken in mice of all different genotypes. (B-E) High magnification confocal retinal images from P0.5 mice of indicated genotypes, all double-stained with anti-FN and anti-VEGF-A. Based on the data shown in Fig. S2, FN is expressed in retinal astrocytes. Furthermore, because VEGF-A displays overlapping distribution patterns with FN, its relationship to astrocytes may be similar to FN. Scale bar: 50 µm. (F) Quantification of FN and VEGF-A expression relative to WT, and the ratio between FN^+^ and VEGF-A^+^ pixels. *n*=6. **P<0.01; ***P<0.001; ns, not significant (*P*=0.065).

Next, we investigated FN and VEGF-A expression in different mouse lines (Fig. 4A-E). Briefly, retinas from P0.5 mice were double stained with anti-FN and anti-VEGF-A and examined by laser confocal imaging. In *Tlx^+/−^* and *Hif2α^f/+^/GFAPCre* mice, FN and VEGF-A were highly expressed, similar to those in WT mice (Fig. 4B-D,F). However, both FN and VEGF-A were far less abundant in *Tlx^+/−^/Hif2α^f/+^/GFAPCre* mice (Fig. 4E,F). It is also noteworthy that the extent of VEGF-A downregulation appeared to be more substantial than fibronectin, as indicated by reduced VEGF-A:FN ratio (Fig. 4F).

### Conclusions and potential mechanisms

In conclusion, our data suggest a developmental mechanism wherein TLX and HIF2α cooperate in retinal astrocytes to support retinal angiogenesis, most likely by maintaining the astrocytes in their proangiogenic progenitor and immature states. Because mature astrocytes are nonangiogenic, accelerated retinal astrocyte maturation in *Tlx^+/−^/Hif2α^f/+^/GFAPCre* mice is a likely contributor to defective retinal vascular development in these mice. Molecularly, TLX/HIF2α dependent FN and VEGF-A expression may be important for retinal angiogenesis. Suppressed retinal vascular morphogenesis in *Tlx^+/−^/Hif2α^f/+^/GFAPCre* mice is consistent with a role of fibronectin and VEGF-A in these developmental processes (Scott et al., 2010; Stenzel et al., 2011).

*Tlx* mRNA expression is hypoxia-dependent in normal neonatal retinas, being highly expressed in the immature astrocytes in the hypoxic avascular regions but downregulated in the mature astrocytes in vascularized areas (Uemura et al., 2006). Such an expression pattern is conducive to its cooperative interaction with HIF2α under physiological hypoxia. We propose that TLX and HIF2α form a feedback mechanism to regulate retinal angiogenesis. In hypoxic areas where angiogenesis is needed, TLX and HIF2α form a protein complex that activates the expression of downstream angiogenic molecules (such as fibronectin, VEGF-A, etc.). As angiogenesis progresses, retinal tissues become vascularized and no further vascular growth is needed. At this point, TLX is downregulated and HIF2α is degraded, both due to increased tissue oxygenation. While this model is proposed based on developmental studies, as a future direction it will be interesting to investigate whether TLX/HIF2α dependent FN deposition and VEGF-A expression also regulate pathological neovascularization.

## MATERIALS AND METHODS

### Mice

All mice were housed and handled according to animal protocols approved by Institutional Animal Care and Use Committee (IACUC) at UConn Health. Production and characterization of *Tlx^+/−^*, floxed *Hif2α* allele, and floxed *Phd2* allele have been described previously (Criscimanna et al., 2013; Shi et al., 2004; Takeda et al., 2006). Mice were in mixed background of C57L/6J:CD1:FVB/N:129 S6 background, where FVB/N background were derived from *GFAPCre* mice (Jackson ImmunoResearch, stock number 004600) (Zhuo et al., 2001), and 129 S6 background was derived from the 129 S6/C57BL/6J hybrid embryonic stems (Criscimanna et al., 2013; George et al., 2007). Overall, C57BL/6J background accounted for >75% in mice used in this study, due to at least two generations of crossing with C57BL/6J. Mice carrying different combinations of alleles were generated by successive steps of breeding, and genotypes were determined by PCR of genomic DNA extracts using previously published methods (Duan and Fong, 2019; Duan et al., 2014; Shi et al., 2004). For staging of early neonatal mice, birth was assumed to occur between midnight and 4 AM; hence newborn mice were considered to be at postnatal day 0.5 (P0.5) by early afternoon. Postnatal ages beyond P1 were rounded to the nearest integer (e.g. P6).

### Whole-mount staining of neonatal retinas and laser confocal microscopy

Eyes were enucleated from euthanized mice and fixed in 4% paraformaldehyde (45 min at room temperature). Retinas were isolated under dissection microscope, and partial radial incisions were made to generate four petals, each attached to the ONH.

Prior to staining, retinas were washed three times in phosphate buffered saline (PBS) and blocked overnight (4°C) in retina staining buffer (RSB, which consists of 1xPBS, 1 mM MgCl_2_, 1 mM CaCl_2_, 1% Triton X-100 and 1% bovine serum albumin). Blocked retinas were incubated with isolectin B4 (IB_4_) to visualize blood vessels, or appropriate primary antibodies. Following incubation with primary antibodies, retinas were washed three times in PBS, and further incubated with secondary antibodies in RSB. All antibodies and other staining reagents are summarized in the Supplemental Information (Table S1 for primary antibodies and Table S2 for secondary antibodies). After overnight incubation at 4°C with IB_4_ or secondary antibodies, retinas were washed three times in PBS and flat-mounted on glass slides. Mounting medium was 50% glycerol in PBS containing 1 mM CaCl_2_ and 1 mM MgCl_2_.

### Analyses of tip cell formation, endothelial cell apoptosis, and EC proliferation

Tip cell analysis was carried out in 8 µm *z*-stack confocal images, generated by Imaris software (Bitplane). Tip cells were identified as IB_4_^+^ cells located at the vascular front, containing large ERG^+^ nuclei, and extending filopodia towards avascular area. Although microglial cells are also double positive for IB_4_ and ERG, most of them are located in avascular areas, away from the vascular front, and their ERG^+^ nuclei are much smaller than those in ECs (Fig. 2E-H).

Apoptotic ECs were detected by double staining of whole-mount retinas with IB_4_ and anti-Caspase 3 (cleaved fragment).

To detect EC proliferation, P6 mice were injected with BrdU intraperitoneally at 120 mg/kg, an hour before euthanasia. Retinas isolated from BrdU-injected mice were treated in 2 mM HCl (1 h at 37°C) before being used for IB_4_ and anti-BrdU double staining. Proliferating ECs were identified as IB4^+^/BrdU^+^ cells.

### Laser confocal imaging and image analyses

Stained retinas were flat-mounted, and images were visualized by laser confocal imaging in Zeiss LSM 880 microscope. Images were recorded with ZEN software (Zeiss). Due to size of retinas, montaged images were generated where necessary. Raw images were further cropped and labeled in Photoshop and/or Adobe Illustrator.

### Quantification of vascular morphogenesis

Quantification of vascular morphogenesis was carried out with the aid of NIH ImageJ. Radial expansion of retinal vasculature was quantified by measuring the distance between the VF and the ONH. Vascular branching morphogenesis was quantified by enumerating the number of branching points in areas just behind the VF, as indicated by red rectangles in Fig. 2A-D. The number of tip cells was counted per 0.5 mm of vascular front. To quantify EC proliferation, the number of BrdU^+^ nuclei was counted per 0.5 mm of IB_4_^+^ microvessels.

### Quantification of immunofluorescence intensity

Quantification of fluorescence signals were carried out with the aid of NIH ImageJ. Pixel values were quantified as the mean grey value in 8-bit grey images. While all images for the same analysis were taken under identical microscope and camera set up, quantification accuracy was further improved by subtracting background values (i.e. mean grey values in blank areas) from the raw values.

### Immunoprecipitation and Western blotting

Retinas were isolated from WT neonatal mice at P4 and homogenized in lysis buffer consisting of 20 mM Tris HCl pH 8, 150 mM NaCl, 1% Triton X-100, 2 mM EDTA, 0.2 mM PMSF (phenylmethylsulfonyl fluoride), and 1x complete protease inhibitor cocktail (Roche). Lysates were incubated with anti-TLX, anti-HIF2α, or nonspecific immunoglobulins (5 µg/ml, 4°C, overnight), followed by 2 h incubations with Protein G magnetic beads (4°C) (Table S1,S2). Protein G beads were collected with a magnetic bar, and precipitated proteins were eluded and denatured by boiling in denaturing loading buffer. Eluted proteins were analyzed by denaturing protein gel electrophoresis and Western blotting.

### Statistical analysis

For statistical analyses, sample sizes (*n*) refer to the number of mice analyzed. *P*-values were calculated by two-tailed Student's *t*-test. *P*-values <0.05 were considered statistically significant. Variations are presented as standard means of error. All statistical analyses were carried out in Excel.

## Supplementary Material

10.1242/biolopen.059684_sup1 Supplementary information
