# Supplementary material for: Tailless and hypoxia inducible factor-2α cooperate to sustain proangiogenic states of retinal astrocytes in neonatal mice
Source: Biol Open. 2023 Jan 10;12(1):bio059684. doi: 10.1242/bio.059684 (PMC9867894; doi:10.1242/bio.059684)
Supplement: Supplementary information [file biolopen-12-059684-s1.pdf]

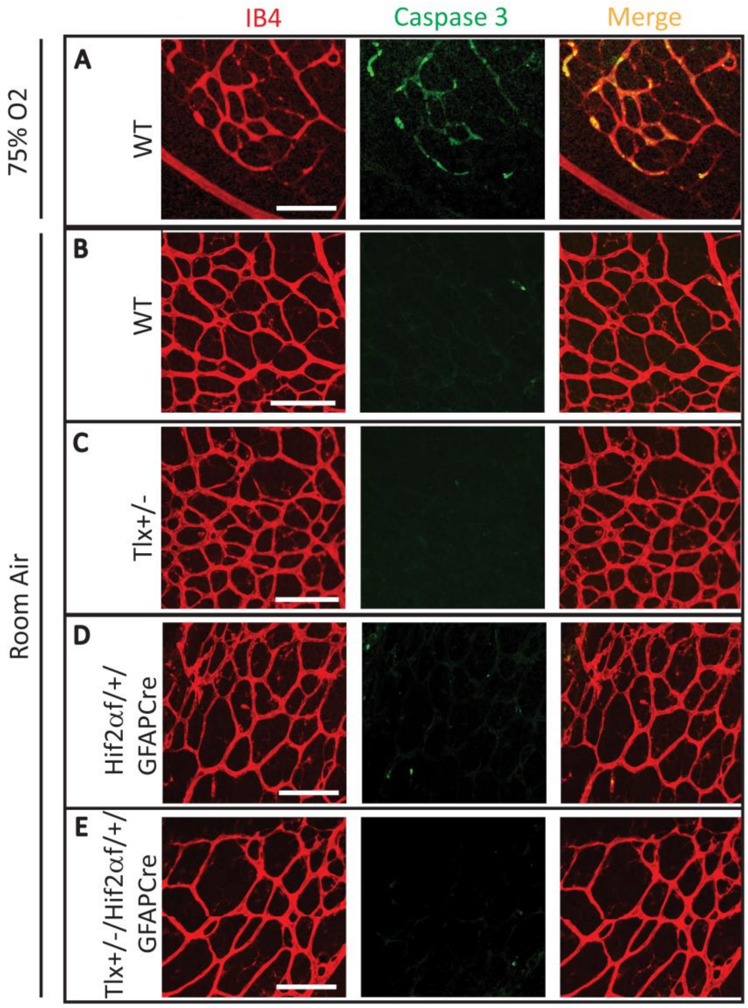

Fig. S1. Apoptosis analyses by anti-Caspase 3 (Asp 175 cleaved fragment) IF-staining.

A. For positive control, wild-type mice treated with 75% oxygen at P7 (14 hours) to induce retinal EC apoptosis (A). Mice in all experimental groups were housed under room air at all times, and retinas were dissected at the equivalent age as the positive control mice (P8) (B-E). In all cases, whole-mount retinas were subject to IF staining with IB_4 and anti-Caspase 3 (Asp175 cleaved), and images were taken from areas at approximately midway between the ONH and the retinal periphery. Scale bars, 100 µm.

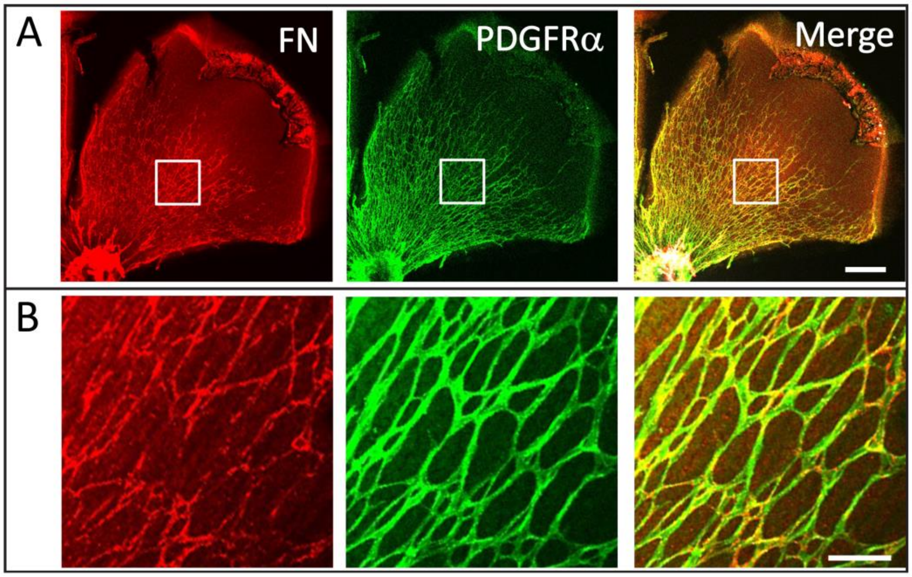

Fig. S2. Expression of fibronectin by retinal astrocyte progenitors in WT mice. Retinas from P0.5 WT mice were IF-stained with anti-FN and anti-PDGFRα, and confocal images were taken at low (A) and high (B) magnifications. Note nearly identical staining patterns for anti-FN and anti-PDGFRα staining (A) and close association between FN^+ and $PDGFRα^+$ structures (B). Since the majority of retinal astrocytes are at progenitor stages in WT mice, these data indicate that retinal astrocyte progenitors express high levels of fibronectin. Bar scale, 200 µm in A, and 50 µm in B.

Biology Open • Supplementary information

Table S1. List of primary antibodies and isolectin B_4.

Line #	Primary antibodies	RRID	Vendor	Catalog #	Final Conc. /dilution	2nd antibodies/ other reagents	Expt.
1	Rat anti-GFAP	AB_ 2532994	Life Technologies (Thermo Fisher)	13-0300	1 µg/ml	Donkey anti-rat IgG-Alexa Fluor®-488	IF staining
2	Rabbit anti-Pax2	AB_ 1603338	Abcam	Ab79389	0.05 µg/ml	Donkey anti-rabbit IgG-Cy3	IF staining
3	Rabbit Anti-Caspase 3 (Asp 175 cleaved)	AB_ 2341188	Cell Signaling	9661S	1 µg/ml	Donkey anti-rabbit IgG Alexa Fluor®-488	IF staining
4	Mouse anti-BrdU -biotin	AB_ 11211335	Millipore-Sigma	MAB3262B	1:400	Alexa Fluor®-488 Streptavidin	IF staining
5	Anti-ERG	AB_ 2630401	Abcam	Ab92513	1 µg/ml	Donkey anti-rabbit IgG-Alexa Fluor®-488	IF staining
6	Goat anti-VEGF-A	AB_ 354506	R&D	AF-493	1:200	Donkey anti-goat IgG Alexa Fluor®-488	IF staining
7	Rabbit anti-Fibronectin	AB_ 447655	Abcam	ab23750	1:500	Donkey anti-rabbit IgG-Cy3	IF staining
8	IB_4-Alexa Fluor®-594		Life Technologies (Thermo Fisher)	I21413	2 µg/ml	Not Applicable	Retina staining
9a	Mouse anti-HIF-2α	AB_ 10000898	Novus Biologicals	NB100-132	5 µg/ml	Protein G magnetic beads	IP
9b	Mouse anti-HIF-2α	AB_ 10000898	Novus Biologicals	NB100-132	1 µg/ml	Goat anti-mouse IgG	Blotting
10a	Rabbit anti-mouse NR2E1 (TLX)	AB_ 2923220	ProSci	22-952	5 µg/ml	Protein G magnetic beads	IP
10b	Rabbit anti-mouse NR2E1 (TLX)	AB_ 2923220	ProSci	22-952	1.67 µg/ml	Goat anti-rabbit IgG-HRP	Blotting
11	Goat anti-ß-actin	AB_ 630836	Santa Cruz Biotechnology	sc-1616	0.1 µg /ml	Donkey anti-goat IgG-HRP	Blotting

Abbreviations: IB_4, isolectin B_4; IF, immunofluorescence; IP, immunoprecipitation; blotting, Western blotting; HRP, horse radish peroxidase.

Biology Open • Supplementary information

Table S2. List of secondary antibodies & protein reagents

Antibodies	Vendor	Catalog #	Final Conc. /dilution	Matching Primary antibody	Expt.	Table S1 line #
Donkey anti-rat IgG-Alexa Fluor®-488	Jackson ImmunoResearch	712-546-153	1 µg/ml	Rat anti-GFAP	IF staining	1
Donkey anti-rabbit IgG-Cy3	Jackson ImmunoResearch	711-166-152	1 µg/ml	Rabbit anti-Pax2	IF staining	2, 7
Alexa Fluor 488-conjugated Streptavidin	Jackson ImmunoResearch	016-540-084	1 µg/ml	Mouse anti-BrdU –biotin	IF staining	4
Donkey anti-rabbit IgG Alexa Fluor®-488	Jackson ImmunoResearch	711-546-152	1 µg/ml	Rabbit anti-Caspase 3	IF staining	3
Donkey anti-goat IgG Alexa Fluor®-488	Life Technologies™ (Thermo Fisher)	A-11055	1 µg/ml	Goat anti-VEGF-A	IF staining	6
Protein G magnetic beads	Invitrogen™ (ThermoFisher)	10003D	5 µl beads /µg IgG	Variable	IP	9a, 10a
Goat anti-mouse IgG-HRP	Jackson ImmunoResearch	115-006-003	0.2 µg/ml	Mouse anti-HIF-2α	Blotting	9b
Goat anti-rabbit IgG-HRP	Jackson ImmunoResearch	111-036-003	0.2 µg/ml	Rabbit anti-mouse NR2E1 (TLX)	Blotting	10b
Donkey anti-goat IgG-HRP	Santa Cruz	sc-2056	0.1 µg/ml	Goat anti-ß-actin	Blotting	11

Abbreviations: IF, immunofluorescence; IP, immunoprecipitation; blotting, Western blotting; HRP, horse radish peroxidase.

Biology Open • Supplementary information
